# Influence of ultrasound on selected microorganisms, chemical and structural changes in fresh tomato juice

**DOI:** 10.1038/s41598-021-83073-8

**Published:** 2021-02-10

**Authors:** Agnieszka Starek, Zbigniew Kobus, Agnieszka Sagan, Barbara Chudzik, Joanna Pawłat, Michał Kwiatkowski, Piotr Terebun, Dariusz Andrejko

**Affiliations:** 1 Department of Biological Bases of Food and Feed Technologies, University of Life Sciences in Lublin, Głęboka 28, 20-612 Lublin, Poland; 2 Department of Technology Fundamentals, University of Life Sciences in Lublin, Głęboka 28, 20-612 Lublin, Poland; 3 Department of Cell Biology, Institute of Biology and Biochemistry, Maria Curie-Skłodowska University, Akademicka 19, 20-033 Lublin, Poland; 4 Institiute of Electrical Engineering and Electrotechnologies, Lublin University of Technology, Nadbystrzycka 38a, 20-618 Lublin, Poland

**Keywords:** Microbiology, Green chemistry, Chemical engineering

## Abstract

The paper presents the possibility of applying ultrasonic technology for inactivation of mesophilic aerobic microorganisms, lactic acid bacteria, coliform bacteria, and yeast with the maintenance of the chemical and structural properties of tomato juice. The research was conducted on fresh tomato juice obtained from the Apis F_1_ variety. Pressed juice was exposed to high power ultrasound and frequency 20 kHz with three operational parameters: ultrasound intensity (28 and 40 W cm^−2^), treatment time (2, 5, and 10 min), and product storage time (1, 4, 7 and 10 days). The temperature of the juice during the sonication ranged from 37 to 52 °C depending on the intensity of ultrasound and time of treatment. Effectiveness of the tested microorganisms eradication in the juice depended on the amplitude and duration of the ultrasound treatment. It was shown that the juice exposed to an ultrasonic field with an intensity of 40 W cm^−2^ for 10 min was microbiologically pure and free from spoilage microorganism even after 10 storage days. No statistically significant differences in pH were found between the untreated juice and the sonicated samples. The ultrasonic treatment was found to change the content of lycopene in small degree (both an increase and a decrease, depending on the processing time) and to induce a small decrease in the vitamin C content. The study suggests that the ultrasonic treatment can be successfully implemented on an industrial scale for the production of not-from-concentrate (NFC) tomato juice.

## Introduction

Sonication is a non-thermal method for food processing having an advantage of preserving fruit juices without unfavourable effects on the nutritional content, sensory properties, and quality of the final product, compared with conventional heat treatments^1–4^. The advantages of ultrasonic treatment are the possibility of reducing microorganisms at a lower temperature than during traditional heat treatment (pasteurization). Ultrasonic treatment of fruit juices had a minimal effect on the content of ascorbic acid and resulted in its better storage stability compared to thermal treatment^5^. Sonication also showed a significant increase in the antioxidant properties (DPPH and TAC) of the apple and grape juice blend compared to the control sample, blanching and high-temperature short-time pasteurization^2^. The combination of ultrasound with pulsed electric field resulted in an increase in the anti-radical activity of grape juice and a higher content of polyphenols, flavonoids, lycopene, anthocyanins and carotenoids^1^. Despite the many advantages of ultrasonic treatment, there are also some disadvantages can affect the quality of fruit juices. These include lipid degradation, colour and viscosity changes, production of off-flavour, and free radical formation^6^.

The mechanism of ultrasonic inactivation of microorganisms is a result of many complex physical processes based on fast-changing mechanical loads, cavitation with a whole range of related phenomena, and in special cases, the so-called cellular resonance. The effect of ultrasound is also attributed to chemical effects related to the generation of free radicals (H^**·**^ and OH^**·**^ via sonochemical reactions) through the decomposition of water inside oscillating bubbles. The application of ultrasonication to microorganisms punctures their cell membranes (thinning of cell membranes) and produces free radicals, and extrusion of the intracellular matrix ultimately kills the microorganisms^6^. Generally, inactivation of microorganisms by ultrasound depends on many factors including ultrasonic power and wave amplitude, temperature, volume of the sample, composition and physical properties of food, type (probiotic, spoilage, or pathogenic microbes) and characteristics of the microorganism (shape, gram negativity or positivity, and growth stage). A limitation in the application of ultrasound in food processing is the problem of heat generation during this process, which can lead to physical, chemical, and biochemical changes in processed raw materials^7–13^. Another restriction of ultrasound is its low efficiency in inactivating spores and yeast. In order to increase the effectiveness of inactivation of these microorganisms, combined techniques are used, for example, the combination of ultrasonication with mild thermal treatment and the use of high-intensity ultrasound^6^. The next limitation is the use of ultrasound on an industrial scale, especially in the case of if high energy is required for the process^14^.

The ultrasound technology has been used for quality enhancement and inactivation of microorganisms in blueberry juice, orange juice, strawberry juice, tomato juice and apple juice. Ultrasonication treatment of juices has been investigated regarding the inactivation of *Escherichia coli*, *Salmonella enterica* serotypes and *Listeria monocytogenes*, *Alicyclobacillus acidiphilus* and *Alicyclobacillus acidoterrestris*, spoilage yeast species^6^ and selected yeasts and moulds (*Penicillium expansum, Aspergillus ochraceus, Rhodotorula *spp.)^15^. Studies carried out on tomato juice revealed 5-log deactivation of yeast (*Pichia fermentans*), with the level of reduction depending on the amplitude and duration of the process^16^. Reduced abundance of *Saccharomyces cerevisiae* was reported by Bermúdez-Aguirre et al.^17^ in investigations on sonication of pineapple, grape, and cranberry juices at 40 °C, 50 °C, and 60 °C for 10 min in the continuous and pulse modes. For example, the grape juice was characterized by total inactivation (7 log) after 10 min of treatment at 60 °C (the continuous mode being more effective). In treatment with high-frequency ultrasound (378 and 583 kHz) with increasing energy densities (up to 250 MJ m^−3^), the content of lycopene and phenolic compounds in tomato juice did not change. However, the high-frequency ultrasound exerted a negative effect on the antioxidant properties of the product^18^.

In acidic food, like tomato juice, spoilage microorganisms are usually found to be restricted to non-spore-forming lactic acid bacteria, yeasts (*Saccharomyces *spp., *Candida *spp.) or molds (*Penicillium*, *Cladosporium*, *Aspergillus*, *Trichoderma*, *Fusarium*). *Bacillus *sp. can also cause spoilage at acidic conditions, especially *Bacillus coagulans* is responsible for the common type of tomato juice spoilage which evident an uncharacteristic acidity^19^. Although *Bacillus coagulans* is a non-pathogenic microorganism, it may cause a food safety hazard due to its ability to increase the pH of acidic foods, to a level that can allow the germination of surviving *Clostridium* spores^20^. The greatest risk to health of consumers are pathogenic microorganisms, the presence of which in the product is not always associated with its organoleptic changes. Due to improper processing, bacteria such as *Escherichia coli*, *Salmonella *spp., *Bacillus cereus*, *Listeria monocytogenes*, *Salmonella *spp., *Staphylococcus aureus* have been found in vegetable juices^21^.

The search for new methods for preservation of freshly pressed juices is becoming an important issue. The ultrasound treatment is reported to be some of the most effective methods in inactivating microorganisms thanks to the physical (cavitation, mechanical, and thermal effects) and chemical (formation of free radicals) action^22^. In addition to inactivation of microorganisms ultrasound treatment also inactivates the enzymes, especially pectin methylesterase and polygalacturonase which are responsible for breaking down of some of the pectins and reducing the viscosity of the juice. The rapid formation and collapse of bubbles are capable of breaking the hydrogen bonds as well as the van der Waals interactions in polypeptide chains. The application of ultrasound alters the secondary and tertiary structure of enzymes and leads to the loss in their biological activity^23^.

The popular one-day juices, classified as “not-from-concentrate” (NFC) juices, are not subjected to enzymatic treatment, clarification, filtration, and pasteurization. No preservatives are added either. Hence, health-enhancing compounds required for the proper function of the human organism are retained in these products^24–26^. However, without the thermal processing step, the low microbiological stability of such products may pose a serious problem for producers. The search for new methods for preservation of freshly pressed juices is becoming an important issue.

Tomato juice deserves special attention as a vegetable product. Lycopene contained in tomatoes exhibits strong antioxidant properties. It has been found that consumption of lycopene present in this raw material is inversely correlated with the risk of development of some cancers and diet-dependent diseases^27^. Tomatoes also contain other bioactive compounds, e.g. β-carotene, vitamins E and C, phenols, organic acids, and flavonoids^28,29^.

Therefore, the aim of the study was to investigate the impact of ultrasound treatment on spoilage microorganisms, chemical properties, and microstructure of freshly pressed tomato juice.

## Materials and methods

### Preparation of tomato juice

Fresh organic tomatoes (*Lycopersicon esculentum* var. Apis F_1_) were purchased in a health food store (Lublin, Poland). The tomatoes were washed with tap water and dried with paper towels. Next, the juice was pressed using a Philips HR1889/70 slow juicer (Amsterdam, the Netherlands). Juice samples were collected for microbiological, chemical, and microscopic analysis.

### Ultrasound treatment

Untreated tomato juice was the control sample. The experimental samples were sonicated using a VC750 Sonics processor (Sonics and Materials Inc., USA) set at 750 W, 20 kHz, 6.8–126 µm, with a 19-mm diameter probe. Sonication was carried out with 46- and 58-µm amplitudes, which correspond to ultrasound intensities of 28 and 40 W cm^−2^. 150-ml tomato juice samples (at an initial temperature of the material of 25 °C) were placed in a 250-ml glass flask and exposed to ultrasound for 2, 5, and 10 min (the ultrasonic probe was immersed in the sample to a depth of 25 mm). The temperature of the juice after the sonication ranged from 37 to 52 °C. The material processed in this way was stored under refrigeration (4 °C) and collected for analyses on days 1, 4, 7, and 10.

### Microbiological evaluation

To assess the effectiveness of the treatment methods used for microbiological decontamination, the juice samples were sent to an accredited food processing microbiological laboratory (Polish Center for Accreditation AB 444) after 1, 4, 7, and 10 days of cold storage. The total numbers of aerobic microorganisms (the method is used for determination of mesophilic lactic acid bacteria that can grow and form colonies on the MRS medium after incubation in aerobic conditions at a temperature of 30 °C for 72 h)^30^, lactic acid bacteria (the method is used for determination of the count of bacteria that are able to grow and form colonies on the VRBL medium after incubation in aerobic conditions at a temperature of 37 °C for 24 h)^31^, coliform bacteria (determination of mould and fungal counts in products with water activity higher than 0.95)^32^, and yeast (the method is used for determination of the counts of moulds and yeasts that are able to grow and form colonies on the DRBC medium in aerobic conditions at a temperature of 25 °C for 5 days in products with water activity higher than 0.95)^33^ were determined by culture or culturing process. The conditions and methods of tested microorganism cultures and colony counts are described in detail in the referenced international ISO standards.

### Chemical and physical analyses

The acidity of the tomato juice was measured using a digital pH Meter 780 (Metrohm, Herisau, Switzerland). 10 ml of the product were placed in a beaker and mixed continuously with a magnetic stirrer. The pH value was measured at 25 ± 0.5 °C. The pH meter was calibrated with buffer solutions at pH 7.0 and 4.0.

The lycopene content was determined with the spectrophotometric method using a Thermo Scientific UV–Vis Helios Omega 3 spectrophotometer (Waltham, Massachusetts, USA). After extraction of the compound with a mixture of acetone with 0.2% BHT, ethanol, and hexane (1:1:2) from the analysed sample, the absorbance of the hexane phase was measured at a wavelength λ = 503 nm^34^.

To determine the content of vitamin C (l-ascorbic acid), the sample was extracted with 2% oxalic acid, filtered, and titrated using Tillmans dye (2,6-dichlorophenolindophenol solution) to obtain a stable pink colour. The amount of the dye expressed in cm^3^ required to oxidize 1 mg of ascorbic acid was used as the 2,6-dichlorophenolindophenol titer^35^.

The colour measurements of juices were performed in triplicate in a colorimeter (4Wave CR30-16; Planeta, Tychy, Poland). The specifications of the colorimeter are illuminant D65, space LAB, and measuring geometry d/8. The measurements were done using a special adapter for fluid measurements. The colour was recorded using CIE-L*a*b* uniform colour space, where L* indicates lightness (from 0–100, black to white), a* denotes red (+)/green (−) value and b* the blue (−)/yellow (+)^36^. Δ*E*, which indicates the magnitude of colour change after treatment, were determined using Eq. (1)

1 $$E=\sqrt{{({L}^{*}-{L}_{0 })}^{2}+{({a}^{*}-{a}_{0 })}^{2}+{({b}^{*}-{b}_{0 })}^{2}}$$

where, L_o_, a_o_ and b_o_ are the colour values of non-sonicated juice samples.

### Microscopic analysis

The microscopic analysis of the fresh tomato juice (control sample) and the sonicated products was carried out using high accuracy, large depth-of-field KEYENCE VHX 950F digital microscope with magnification ability from 100 × to 1000 ×, CMOS camera and without the module of light polarization^37^. After each ultrasonic treatment, samples of juice were mixed and triplicate samples of 1 ml volume from the control and the treated lots were positioned on the surface of the microscopic slide. Observations were carried out after protecting the sample with the cover glass and placing it on the manipulation stage.

### Statistical analyses

The obtained results were statistically analysed with Statistica software^38^ via analysis of variance (ANOVA). The significance of differences between the evaluated mean values (in figures) was analysed with the Tukey test at a significance level of p < 0.05. The tables present the mean values with standard deviations, while the graphs present mean values and whiskers representing standard deviations. The results of the microbiological analyses and chemical properties are presented as average values of five and three measurements in each sample, respectively.

## Results and discussion

### Microbiological evaluation after sonication

Applicable microbiological criteria for foodstuffs, including unpasteurized fruit and vegetable juices are included in the Regulation of European Commission (EC) No 2073/2005 of 15 November 2005^39^. The only safety criterion specified in this regulation is the absence of *Salmonella* and the limit of *E. coli* bacteria in unpasteurized fruit and vegetable juices. There is a lack of regulations for the permissible quantity of other microorganisms in these types of products. The regulation applicable in Poland defines the maximum number of aerobic mesophilic microorganisms at the level of 10^3^–10^4^ CFU/ml only in pasteurized fruit and vegetable juices^40^. The data available in the literature on the total number of microorganisms in fresh fruit and vegetable juices differ greatly (from 2 to 7 log_10_ CFU/ml) depending on the sanitary regime of the process of juice production, the method of vegetable cultivation, variety, and storage conditions^41,42^. Studies have shown that the number of total aerobic microorganisms higher than 4 log_10_ CFU/ml is responsible for juice spoilage.

In our study, the microbiological analysis of the untreated tomato juice (control) showed its insufficient microbiological quality after the first day of storage. At that time, the average level of contamination of these samples with mesophilic aerobic microorganisms, lactic acid bacteria, coliform bacteria, and yeast was 6.1 log_10_ CFU/g, 4.2 log_10_ CFU/g, 2.4 log_10_ CFU/g and 3.7 log_10_ CFU/g, respectively. A similar total number of aerobic microorganisms (6.3 log_10_ CFU/g) was recorded after the 4-day storage of the juice. Their number on the consecutive days increased to 6.5 log_10_ CFU/g (day 7) and 6.8 log_10_ CFU/g (day 10). The number of lactic acid bacteria was 4.2 log_10_ CFU/g after 4 days of storage and reached 4.6 log_10_ CFU/g and 5.6 log_10_ CFU/g after days 7 and 10, respectively. The coliforms multiplied to the level of 2.4 log_10_ CFU/g on storage day 4, and their count further increased by 0.3 and 0.4 log_10_ CFU/g on day 7 and 10, respectively. Similarly, the content of yeast colonizing the tomato juice on storage day 4 reached a value of 3.8 log_10_ CFU/g, which increased to 3.9 log_10_ CFU/g after 7 days of storage. In the final stage of storage, their abundance was estimated at 3.9 log_10_ CFU/g.

**Figure 1:**
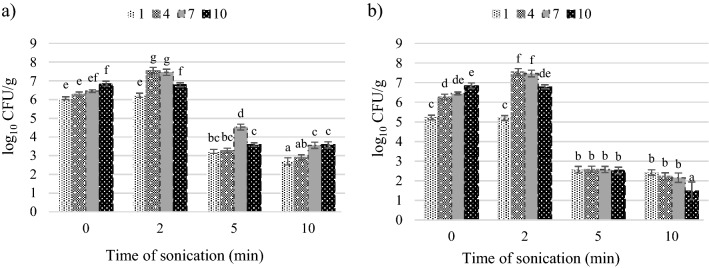
Effect of ultrasound intensity and sonication time on the total aerobic mesophilic bacteria count: (**a**) intensity 28 W cm^−2^, (**b**) intensity 40 W cm^−2^.

The process of tomato juice sonication contributed to the reduction of the number of the analysed microorganisms; however, this effect was associated with the duration of treatment and the ultrasound intensity. The ultrasonic treatment of tomato juice with an intensity of 28 and 40 W cm^−2^ for 2 min resulted in an increase in the total number of microorganisms by maximum of 1.3 log_10_ CFU/g (Fig. 1).

The multiplication of microorganisms was stimulated by mild ultrasound treatment. Many authors have shown that the effect of ultrasound on the proliferation of microbial cells depends on the intensity and duration of the treatment. While the high intensity of ultrasound treatment causes irreversible cell damage, low-intensity ultrasound, causing steady cavitation, stimulates the proliferation of microbial cells and their increased metabolic activity or even the production of the desired product^43,44^. It was investigated that low-intensity ultrasound can cause increased permeability of *S. cerevisiae* cell membranes, resulting in increased Ca^2+^ content in the cytoplasm and enhanced cell proliferation^45^. In addition to increased cell proliferation, ultrasound with an appropriately selected application regimen can induce genetic mutations and is used for screening for mutants showing the desired characteristics, e.g. with increased synthesis of some products^46,47^.

**Figure 2:**
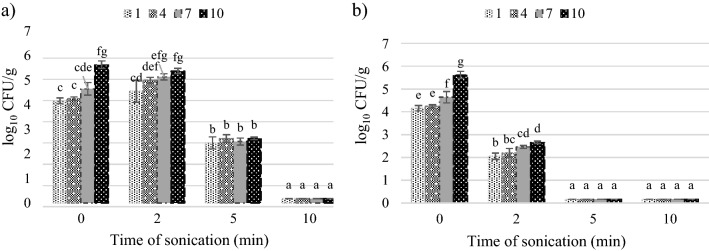
Effect of ultrasound intensity and sonication time on lactic acid bacteria count: (**a**) intensity 28 W cm^−2^, (**b**) intensity 40 W cm^−2^.

The lower intensity ultrasound after 2 min of treatment also slightly increased the number of lactic acid bacteria which form colonies at 30 °C in a solid selective medium (MRS at pH 5.7), whereas the intensity of 40 W cm^−2^ reduced their number to about 3 log_10_ CFU/g (Fig. 2).

**Figure 3:**
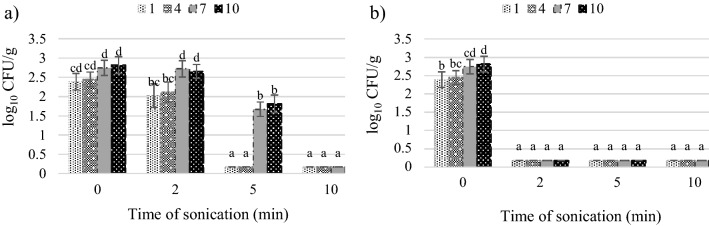
Effect of ultrasound intensity and sonication time on the coliform bacteria count: (**a**) intensity 28 W cm^−2^, (**b**) intensity 40 W cm^−2^.

The number of coliform bacteria was not changed after 2 min treatment with 28 W cm^−2^ ultrasound and decreased by 1.8–2.6 log_10_ CFU/g after 2 min of 40 W cm^−2^ (Fig. 3).

**Figure 4:**
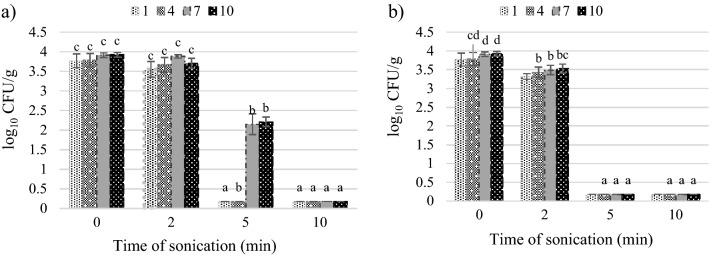
Effect of ultrasound intensity and sonication time on the total yeast count: (**a**) intensity 28 W cm^−2^, (**b**) intensity 40 W cm^−2^.

Yeasts colonizing the tomato juice exhibited low sensitivity to the 2 min of ultrasound exposure. The treatment reduced their level by 0.02–0.2 log_10_ CFU/g (intensity of 28 W cm^−2^) and 0.3–0.5 log_10_ CFU/g (intensity of 40 W cm^−2^) (Fig. 4). From these results it is visible, that the 2 min ultrasound treatment with 28 and 40 W cm^−2^ is insufficient for effective microbial load reduction in the tomato juice. A good result is that the number of coliform bacteria was not increased by a shorter time of treatment and was successfully eradicated by 40 W cm^−2^ ultrasound because in large quantities they can be hazardous to health^48^.

After the 5 min of sonication, there was a significant reduction in the total number of microorganisms, compared to the control. In terms of the total number of aerobic microorganisms, the number of colony-forming units after the storage period decreased by 3.2 log_10_ CFU/g in the treatment with the ultrasound intensity of 28 W cm^−2^ and by 4.3 log_10_ CFU/g at the intensity of 40 W cm^−2^. In turn, the number of lactic acid bacteria was reduced by 1.6–3.0 log_10_ CFU/g at the intensity of 28 W cm^−2^ and to < 1 log_10_ CFU/g, i.e. below the limit of quantification, at the intensity of 40 W cm^−2^. The presence of coliform bacteria was detected only on storage day 4 (1.7 log_10_ CFU/g) and day 7 (1.8 log_10_ CFU/g) in the treatment of the tomato juice with the lower ultrasound intensity. Regardless of the duration of storage, the numbers of yeasts at both intensities were below 10 CFU/g, i.e. below the limit of quantification.

The extension of the duration of the ultrasonic treatment to 10 min ensured microbiological purity of the tomato juice, which was free of microorganisms responsible for the deterioration of the product even after 10 days of storage. In comparison with the control sample, the total number of microorganisms was reduced by 2.9–3.4 CFU/g at the ultrasound intensity of 28 W cm^−2^ and by 2.8–5.4 CFU/g at 40 W cm^−2^. The number of the other microorganisms was reduced to an undetectable level, i.e. < 10 CFU/g. The number of CFU/ml of mesophilic aerobic microorganisms which survived the treatment for 10 min is acceptable according to the regulation, even in pasteurized fruit and vegetable juices^40^. The research methods used by us did not allow for the identification of the species of remaining microorganisms extremely resistant to sonication.

The effects of ultrasound treatment are not universal and depend on the juice being treated^44^. To date, many attempts have been made to inactivate microorganisms introduced into juice, e.g. *E. coli* O157: H7, *Salmonella *spp., or *S. cerevisiae* in apple juice^49^. Approximately 7-log reduction of the number of *P. fermentans* yeasts was noted in infected tomato juice subjected to sonication (maximum temperature: 45 °C, time: 10 min)^16^. The reduction of the number of cells of selected yeasts and moulds (*A. ochraceus* 318, *P. expansum* 565, *Rhodotorula *sp. 74) by ultrasound treatments at 60 °C applied for 3, 6, and 9 min ranged from 3.556 to 5.934 log units. It depended on the initial number of the yeasts and moulds in clear juices and nectars from apple, blueberry, and cranberry juice concentrate before the treatment^15^. As demonstrated by Alighourchi et al.^50^, lower amplitude levels (50 and 75%) did not inactivate *E. coli* and *S. cerevisiae* significantly (< 1.5 log reduction), while treatment at a 100% amplitude level for 15 min reduced their population by 3.47 and 1.86 log CFU/ml, respectively. The inactivation of microbial cells may have resulted from the combination of physical and chemical mechanisms occurring during the sonication process. The generation of free radicals and H_2_O_2_ in microbial cells induced by sonication supporting the inactivation of organisms was described by Oyane et al.^51^. The process may not be as effective in inactivation of yeast cells, since their rigid cell wall prevents direct destruction by ultrasound. Other authors investigating the effect of ultrasound on yeast cells did not observe cell rupture and release of intracellular proteins as a direct result of sonication^16^. Unfortunately, due to the high content of the total number of microorganisms, only the higher intensity of the sonication process ensured the high microbiological quality of the product and its suitability for consumption.

The main advantage of sonication during inactivation of microorganisms is the reduction of the processing time to achieve the same lethal effect at moderate temperatures. Lopez-Malo et al.^52^ observed significant reductions in the decimal reduction time for *S. cerevisiae* at 45 °C from 739 min (heat treatment alone) to 22 min, when the heat was combined with ultrasonic treatment. Similarly, Nguyen and Mittal^53^ reported that at moderate temperature (47 °C) only 0.9 log microbial reduction was achieved after 30 min of heating. The application of ultrasound for inactivation of microorganisms in tomato juice is a more effective technique than the use of a pulse electric field. Only marginal effects against microbial inactivation (about 1.4 log reduction) were observed by Nguyen and Mittal^53^ during combining heat treatment (50 °C) with PEF (20 pulses at 80 kV/cm). In comparison in our research, the ultrasound intensity of 40 W cm^−2^ allowed to reduce the total number of microorganisms by 2.8–5.4 log deepening on time of treatment.

### Physicochemical analysis

#### pH

Acidity is an important parameter of juice quality. It affects not only the taste of the juice but also the possibility of the development of microorganisms. In foods, it is not only a function of the type and concentrations of acids present but also of the concentration of ionized acid counterparts (their conjugated bases)^54,55^. The effect of sonication on pH of juice have been reported also by other authors—Bhat et al.^56^, who analysed kasturi lime juice (pH 3.99–4.00), Abid et al.^57^, who sonicated apple juice for 30, 60, and 90 min (pH 3.99–4.00), and Zafra-Rojas et al.^58^, who treated purple cactus pear juice with ultrasound intensity of 14–28 W cm^−2^ for 10 to 25 min (pH 5.00–5.11). Sonication carried out in an ultrasonic bath at a frequency of 28 kHz and a constant temperature of 20 °C did not change the pH value of grapefruit juice, compared to control samples^59^.

The results of the assessment of the impact of sonication on the tested parameters are presented in Table 1. Immediately after pressing, the fresh tomato juice (control) was characterized by the following parameters: pH—3.91 ± 0.04, lycopene content—3.33 ± 0.02 mg/100 g, and vitamin C content—12.27 ± 0.04 mg/100 g. No statistically significant differences in this parameter were observed in the freshly pressed juice between day 1 (3.91) and day 4 of cold storage (3.94) (p < 0.69). Differences were found only after 7 days of storage (4.08). On day 10, the product deteriorated due to the appearance of mould. The pH value in the sonicated juice stored for one day was slightly higher than that in the untreated juice, especially in the product treated with the ultrasound intensity of 40 W cm^−2^ for 5 and 10 min. In most cases, the storage time had an effect on the pH value of the tomato juices. The highest values of this parameter, i.e. 4.55 and 4.52, were recorded after 10 days of cold storage of samples treated with ultrasound intensity of 28 and 40 W cm^−2^ for 10 min, respectively.

**Table 1:** Effect of sonication parameters on the chemical properties of tomato juice stored for 10 days.

Chemical parameters	Time of sonication (min)	Intensity (W cm^−2^)	Time storage (days)			
			1	4	7	10
Acidity (pH)	0 (control)		3.91 ± 0.00Aa	3.94 ± 0.06Aab	4.08 ± 0.04Ab	Unmeasured
	2	28	4.33 ± 0.01Ba	4.34 ± 0.01Ba	4.39 ± 0.01Bb	4.40 ± 0.03Ab
	5		4.33 ± 0.01Ba	4.35 ± 0.00Ba	4.44 ± 0.01Bb	4.46 ± 0.05Ab
	10		4.35 ± 0.04Ba	4.40 ± 0.08Bab	4.42 ± 0.01Bab	4.56 ± 0.06Ab
	2	40	4.35 ± 0.05Ba	4.39 ± 0.02Ba	4.42 ± 0.01Ba	4.46 ± 0.01Aa
	5		4.36 ± 0.00Ba	4.37 ± 0.01Ba	4.37 ± 0.07Ba	4.51 ± 0.06Ab
	10		4.37 ± 0.00Ba	4.39 ± 0.04Ba	4.43 ± 0.04Ba	4.52 ± 0.09Aa
Lycopene (mg/100 g)	0 (control)		3.33 ± 0.00Bb	3.32 ± 0.01Bab	3.19 ± 0.02Aa	Unmeasured
	2	28	3.36 ± 0.02Ba	3.36 ± 0.02Ba	3.35 ± 0.10Ba	3.33 ± 0.01Ba
	5		3.42 ± 0.01Ca	3.41 ± 0.00Ca	3.41 ± 0.01Ca	3.36 ± 0.03Ba
	10		3.44 ± 0.05Cab	3.44 ± 0.04Cab	3.41 ± 0.08Cab	3.33 ± 0.80Ba
	2	40	3.67 ± 0.00Eb	3.26 ± 0.03Aa	3.66 ± 0.05Eb	3.55 ± 0.02Cb
	5		3.62 ± 0.01Da	3.32 ± 0.01Ba	3.60 ± 0.04Da	3.53 ± 0.01Ca
	10		3.24 ± 0.03Ab	3.33 ± 0.01Bb	3.21 ± 0.02Aa	3.14 ± 0.05Aa
Ascorbic acid (mg/100 g)	0 (control)		12.27 ± 0.04Cc	11.70 ± 0.02Cb	9.09 ± 0.12Aa	Unmeasured
	2	28	12.27 ± 0.08Ca	12.27 ± 0.05Ea	12.27 ± 0.03Fa	12.30 ± 0.01Da
	5		12.11 ± 0.02Ca	12.02 ± 0.03Da	11.99 ± 0.08Ea	11.83 ± 0.25Ca
	10		11.41 ± 0.06Ba	11.34 ± 0.00Ba	11.32 ± 0.02Ca	11.30 ± 0.10Ba
	2	40	11.56 ± 0.03Bb	11.44 ± 0.05Bab	11.42 ± 0.03Da	11.40 ± 0.01Ba
	5		11.39 ± 0.04Bb	11.35 ± 0.04Bab	11.31 ± 0.01Cab	11.21 ± 0.01Ba
	10		9.97 ± 0.13Ab	9.82 ± 0.08Ab	9.72 ± 0.02Bab	9.64 ± 0.01Aa

The results are expressed as a mean ± standard error.

Average values in the column marked with the same capital letter are not statistically significantly different (p < 0.05).

Average values in the raw small marked with the same small letter are not statistically significantly different (p < 0.05).

#### Lycopene

Tomatoes and tomato products are an important source of many compounds with high biological activity, with the largest content of carotenoids, including the health-promoting lycopene. This pigment is present in varying amounts in both the skin and the flesh of tomato fruits and is responsible for the characteristic deep red colour of ripe fresh and processed tomatoes^60,61^. Irrespective of the intensity and duration of the ultrasound applied (in most cases), the content of lycopene in the sonicated tomato juice was unchanged. In turn, the 2 min treatment (intensity 40 W cm^−2^) resulted in a 9% increase in the lycopene content after the first day of storage, compared with the control sample. However, the further extension of the ultrasonic treatment time to 10 min resulted in a small but statistically significant decrease in the lycopene content, compared with the control. The storage time did not contribute to the reduction of this pigment after the sonication process, with the exception of the untreated tomato juice samples and those sonicated for 10 min (intensity 40 W cm^−2^) and stored for 7 or 10 days. An increase in the total carotenoid content was also observed in orange juice samples^62^ treated with ultrasound for 1–30 min, compared with an untreated product. As suggested by Guerrouj et al.^62^, this is caused by the mechanical disruption of cell walls, which may increase the amount of total carotenoid in the research material.

#### Ascorbic acid

Vitamin C is a thermolabile compound; hence, the ultrasonic treatment, in which a large part of the acoustic energy is converted into heat energy (increase in the temperature of the sonicated medium), can accelerate the decomposition of this biologically active food ingredient. The freshly pressed tomato juices exposed to ultrasound had lower ascorbic acid content than the control samples. On the first day of storage, the content of this compound after the treatment with the ultrasound intensity of 40 W cm^−2^, especially after the 10 min treatment, was by approx. 19% lower than in the freshly pressed juice. Substantially lower losses (approx. 7%) were observed in the variant with the ultrasound intensity of 28 W cm^−2^ and sonication time of 5 and 10 min. Vitamin C contained in the analysed products was further degraded during storage, both in the control sample and in the juices treated with ultrasound intensity of 40 W cm^−2^. Adekunte et al.^63^ reported degradation of ascorbic acid during the sonication process, although the analysed tomato juice was placed in a vessel with a water jacket with a temperature of 25 ± 0.5 °C (the temperature of the sonicated juice ranged from 32 to 45 °C). The content of this vitamin in the material treated for 10 min decreased from 14.5 mg/100 ml (control) to 9.8 mg/100 ml. In contrast, Cheng et al.^64^ found that ultrasonic treatment carried out at low temperatures increased the content of ascorbic acid in guava juice, compared with the non-sonicated control sample, due to the elimination of dissolved oxygen, which is necessary for the degradation of this compound during cavitation.

#### Colour

The effect of sonication on the colour of tomato juice was dependent on the intensity of the ultrasound and the duration of the treatment (Table 2).

**Table 2:** Effects of ultrasound processing on colour attributes.

Time of sonication (min)	Intensity (W cm^−2^)	Colour attributes			ΔE
		L*	a*	b*	
0 (control)		31.75 ± 0.19A	10.91 ± 0.12A	6.47 ± 0.43A	–
2	28	32.02 ± 0.84A	11.05 ± 0.18A	6.61 ± 0.15A	0.33 ± 0.12A
5		32.49 ± 0.54A	11.02 ± 0.06A	6.90 ± 0.22A	0.86 ± 0.23A
10		32.65 ± 0.17A	11.07 ± 0.13A	7.34 ± 0.18B	1.26 ± 0.21B
2	40	32.16 ± 0.41A	11.20 ± 0.04A	6.69 ± 0.22A	0.55 ± 0.16A
5		33.11 ± 0.18B	11.07 ± 0.03A	7.37 ± 0.19B	1.64 ± 0.32B
10		34.01 ± 0.19C	11.06 ± 0.06A	7.58 ± 0.18B	2.52 ± 0.30C

Values with different letters in the same column are significantly different from each other.

A statistically significant increase in L* values (lightness) was observed only for samples treated at 40 W cm^−2^ for 5 and 10 min. No significant changes were observed in a* (redness). In the case of the b* (yellowness), statistically significant differences were observed for the juice sonicated for 10 min at both tested intensities and for the juice sonicated for 5 min at the intensity 40 W cm^−2^. Higher lightness in treated samples can be attributed to inactivation polyphenols oxidase (PPO) by sonication and heat^65^. A similar increase in lightness was observed by Lee et al.^66^ during the heat and ultrasonic treatment of apple cider. Results regarding colour values were in agreement with earlier findings of Zhang et al.^67^ who also reported an increase in lightness and yellowness and no change in redness during the sonication of tomato juice.

ΔE values increased from 0.33 to 2.52 indicating visual colour variation. Differences in perceivable colour can be noticed if ΔE > 1. This corresponds well with the results of the statistical analysis. Statistically significant differences between the sonicated juices were found only when the value of the ΔE parameter was higher than 1.

#### Microstructure analysis

**Figure 5:**
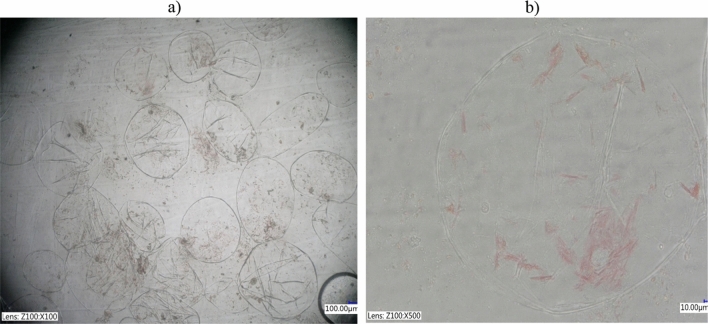
Microstructure of freshly pressed tomato juice at magnification.

**Figure 6:**
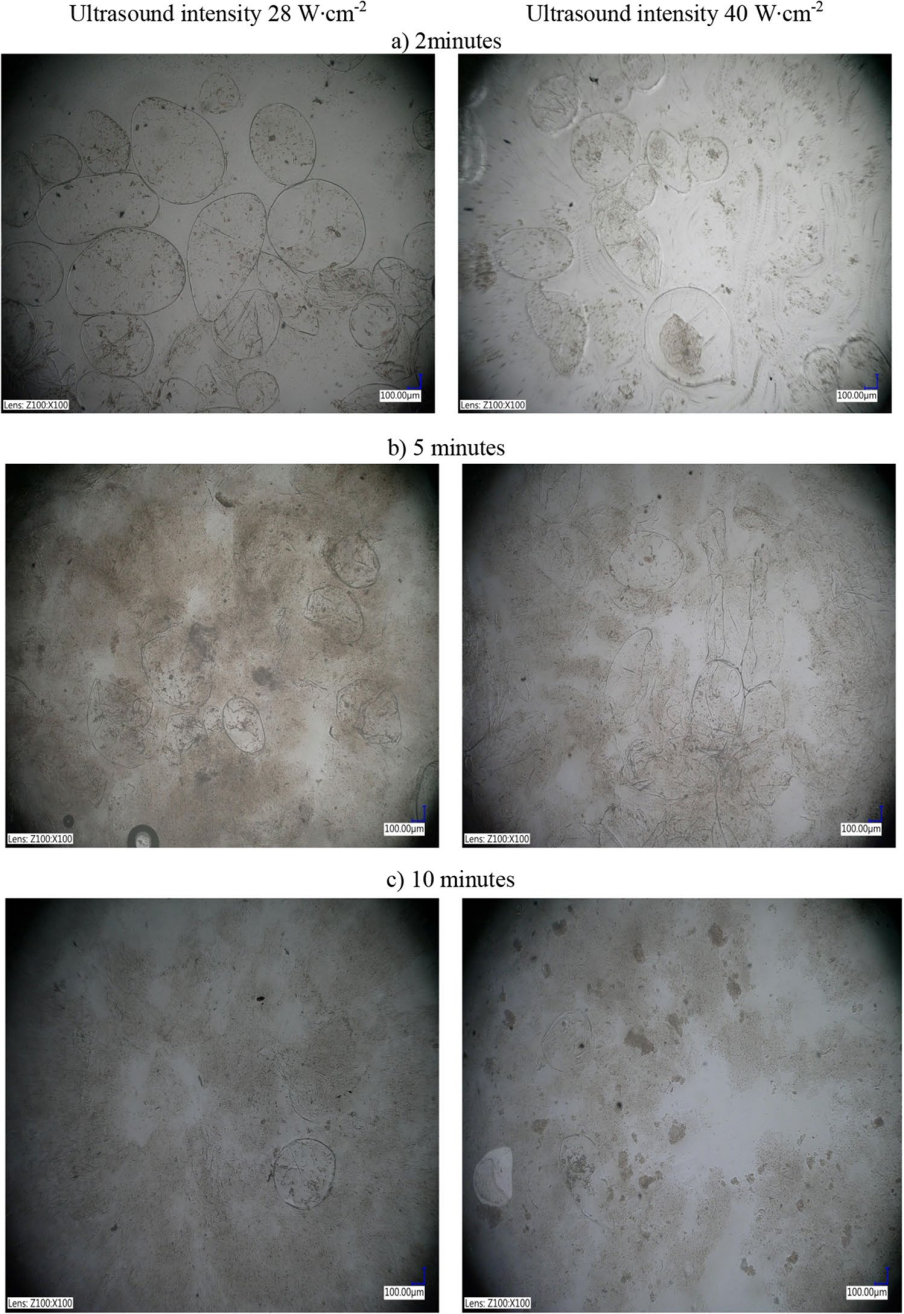
Microstructure of tomato juice sonicated at ultrasound intensity of 28 and 40 W cm^−2^ for: (**a**) 2 min, (**b**) 5 min, and (**c**) 10 min.

Figures 5 and 6 show selected and most distinctive digital microscopy images of the microstructure of the tomato juice. The untreated tomato juice (control sample) contained undamaged oval-shape cells with intact organelles containing carotenoids, including clearly visible “needle-like” light pink lycopene crystals, corresponding to the aggregations of this unsaturated aliphatic hydrocarbon with elongated chemical structure (Fig. 5a,b).

Figure 6a–c shows digital microscopy images (100 × magnification) of tomato juice exposed to ultrasound at an intensity of 28 and 40 W cm^−2^ for 2, 5, and 10 min.

The product sonicated at 28 and 40 W cm^−2^ for 2 and 5 min contained cell clusters consisting of several cells. In spite of apparently intact cell wall, the cells in the clusters were slightly smaller and showed signs of plasmolysis (shrinkage and detachment of the protoplast from the cell wall), compared with the control. Lycopene crystals were visible inside intact chromoplasts. Especially, after 2 min at 28 W cm^−2^ sonication treatment seemed to have a mild impact on the cellular structure. In contrast, the tomato juice sonicated for 10 min (especially at 40 W cm^−2^) did not have intact cells (stained carotenoids were released from chromoplasts and cells into the amorphous part of the juice). Elongated aggregations of lycopene crystals were disrupted and not visible in the observation area. Instead, irregular dark colour aggregations of smaller size fractions could be noticed. Microscopically dense and solid fraction of juice structure was homogenized and aggregated. Space between large aggregation zones was filled with the thin almost transparent liquid phase.

Anese et al.^68^ confirmed that a longer ultrasound process was accompanied by loss of cell integrity of tomato pulp. Microscopic images of samples treated for 15 and 30 min showed both intact cells containing carotenoid crystals and damaged cells whose internal components were present inside and were suspended in the tomato homogenate. Complete disintegration of cells and cell organelles was observed in tomato pulp treated with ultrasound for 60 min (the material composed of disintegrated cells was evenly distributed in the sample).

## Conclusion

The present study showed that properly selected parameters of the sonication process contributed to the high and stable microbiological quality of tomato juice. The ultrasound sonication at the intensity of 40 W cm^−2^ for 5 min and 28 W cm^−2^ for 10 min yielded a microbiologically pure product devoid of microorganisms involved in spoilage even after 10-day storage. The number of the analysed microorganisms was reduced to an undetectable level (< 10 CFU/g). The pH values and lycopene content after sonication (in most cases) did not differ statistically significantly in comparison with the untreated product. There was a slight decrease in the content of ascorbic acid in the tomato juice. The microscopic analysis revealed a different degree of juice homogenization related to the intensity of the ultrasonic field applied. The results suggest that ultrasonic treatment can be successfully employed to produce high-quality tomato juice with a long shelf life.
